# Temporal Changes in Obesity-Related Medication After Bariatric Surgery vs No Surgery for Obesity

**DOI:** 10.1001/jamasurg.2023.0252

**Published:** 2023-05-24

**Authors:** Joonas H. Kauppila, Sheraz Markar, Giola Santoni, Dag Holmberg, Jesper Lagergren

**Affiliations:** 1 Upper Gastrointestinal Surgery, Department of Molecular Medicine and Surgery, Karolinska Institutet, Karolinska University Hospital, Stockholm, Sweden; 2 Department of Surgery, Oulu University Hospital and University of Oulu, Oulu, Finland; 3 Nuffield Department of Surgery, University of Oxford, Oxford, United Kingdom; 4 School of Cancer and Pharmaceutical Sciences, King's College London and Guy's and St Thomas' NHS Foundation Trust, London, England

## Abstract

**Importance:**

Bariatric surgery can resolve hyperlipidemia, cardiovascular disease, and diabetes, but the long-term postoperative trajectories of medications for these conditions are unknown.

**Objective:**

To clarify the long-term use of lipid-lowering, cardiovascular, and antidiabetic medication after bariatric surgery compared with no surgery for morbid obesity.

**Design, Setting, and Participants:**

This population-based cohort study took place in Sweden (2005-2020) and Finland (1995-2018) and included individuals diagnosed with obesity. Analysis took place between July 2021 and January 2022.

**Exposures:**

Bariatric surgery (gastric bypass or sleeve gastrectomy) patients using lipid-lowering, cardiovascular, or antidiabetic medication were compared with 5 times as many control patients with an obesity diagnosis treated with no surgery, matched for country, age, sex, calendar year, and medication use.

**Main Outcomes and Measures:**

Proportions with 95% CIs of lipid-lowering, cardiovascular, or antidiabetic medication.

**Results:**

A total of 26 396 patients underwent bariatric surgery (with gastric bypass or sleeve gastrectomy) (17 521 [66.4%] women; median [IQR] age, 50 [43-56] years) and 131 980 matched control patients (87 605 [66.4%%] women; median [IQR] age, 50 [43-56] years) were included. The proportion of lipid-lowering medication after bariatric surgery decreased from 20.3% (95% CI, 20.2%-20.5%) at baseline to 12.9% (95% CI, 12.7%-13.0%) after 2 years and 17.6% (95% CI, 13.3%-21.8%) after 15 years, while it increased in the no surgery group from 21.0% (95% CI, 20.9%-21.1%) at baseline to 44.6% (95% CI, 41.7%-47.5%) after 15 years. Cardiovascular medications were used by 60.2% (95% CI, 60.0%-60.5%) of bariatric surgery patients at baseline, decreased to 43.2% (95% CI, 42.9%-43.4%) after 2 years, and increased to 74.6% (95% CI, 65.8%-83.4%) after 15 years, while it increased in the no surgery group from 54.4% (95% CI, 54.3%-54.5%) at baseline to 83.3% (95% CI, 79.3%-87.3%) after 15 years. Antidiabetic medications were used by 27.7% (95% CI, 27.6%-27.9%) in the bariatric surgery group at baseline, decreased to 10.0% (95% CI, 9.9%-10.2%) after 2 years, and increased to 23.5% (95% CI, 18.5%-28.5%) after 15 years, while it increased in the no surgery group from 27.7% (95% CI, 27.6%-27.7%) at baseline to 54.2% (95% CI, 51.0%-57.5%) after 15 years.

**Conclusions and Relevance:**

In this study, undergoing bariatric surgery was associated with a substantial and long-lasting reduction in the use of lipid-lowering and antidiabetic medications compared with no surgery for obesity, while for cardiovascular medications this reduction was only transient.

## Key Points

**Question:**

What are the long-term trajectories of lipid-lowering, cardiovascular, and antidiabetic medications after bariatric surgery vs no surgery for obesity?

**Findings:**

In this population-based cohort study of 26 396 patients who underwent bariatric surgery and 131 980 matched controls with obesity in Sweden and Finland between 1995 and 2020, the proportions of users of lipid-lowering, cardiovascular, and antidiabetic medication in the long term were considerably lower compared with patients with obesity not undergoing bariatric surgery.

**Meaning:**

Bariatric surgery may reduce the use of lipid-lowering, cardiovascular, and antidiabetic medications in the long term compared with no surgery for obesity.

## Teaser

This cohort study aims to clarify the long-term trajectories of lipid-lowering, cardiovascular, and antidiabetic medication in patients who have undergone bariatric surgery compared with matched patients who received no surgery for morbid obesity.

## Introduction

Bariatric surgery decreases the prevalence of obesity-related diseases,^1,2^ contributing to longer life expectancy and increasing use of such surgery in patients with morbid obesity.^3,4,5,6^ Yet, it is unclear how bariatric surgery influences the long-term use of medications for obesity-related diseases, ie, hyperlipidemia, cardiovascular disorders, and diabetes.^2,7,8^ No study has examined the trajectories of medication use beyond 8 years following bariatric surgery. Furthermore, only one of these studies with 6 years of follow-up used a population-based design, where selection bias is avoided.^7^ The complete and high-quality national data from the Nordic countries allow for large population-based studies with long and complete follow-up. We aimed to clarify the long-term trajectories of lipid-lowering, cardiovascular, and antidiabetic medication in patients who have undergone bariatric surgery compared with matched patients who received no surgery for morbid obesity in a Swedish and Finnish study.

## Methods

### Design

This population-based cohort study included all patients with any obesity diagnosis in Sweden or Finland. Data came from the nationwide patient registries, medication registries, and death registries in both countries (presented below). The total study period was from 1995 to 2020 but took place from 2005 to 2020 in Sweden and from 1995 to 2018 in Finland with years depending on the initiation of the medication registries and the updates of the registries when data were retrieved. The reporting to these registries is mandatory by law and is linked to the remuneration of the hospitals, making the registries highly complete.^9^ Personal identity numbers are used by all Swedish and Finnish residents, enabling accurate linkages and merging of information for each individual.^7,10^ The study was approved by Regional Ethical Review Board in Stockholm, relevant data inspectorates, and governmental agencies in Sweden and Finland. Informed consent is not required for registry studies in Sweden and Finland. We followed the Strengthening the Reporting of Observational Studies in Epidemiology (STROBE) reporting guideline.

### Data Sources

The patient registries, namely the Swedish National Patient Register in Sweden and the Care Register for Health Care in Finland, provided information on diagnoses and operations related to hospital admissions, with high positive predictive values.^10,11^ We have evaluated bariatric surgery data of the Swedish National Patient Register in a separate validation study of 938 records, showing a positive predictive value of 97.0%.^12^

The medication registries, namely the Prescribed Drug Register in Sweden and the Finnish Prescription Register in Finland had data on the number, type, and dose of all prescribed and dispensed medications. The data have high completeness and accuracy due to direct and automatic (computerized) issuing from the pharmacies to the registries.^13,14^ Lipid-lowering, cardiovascular, and antidiabetic medications are only available through prescriptions and are thus fully covered by these registries.

The death registries, namely The Swedish Cause of Death Register in Sweden and Cause-of-Death Register in Finland provided data on dates of all-cause mortality with 100% completeness.^9^

### Exposure

Within the cohort of patients with at least 1 inpatient or outpatient obesity diagnosis as a primary or secondary diagnosis, the exposed group was patients who underwent primary bariatric surgery with gastric bypass or sleeve gastrectomy (bariatric surgery group). Gastric banding, duodenal switch, and other types of bariatric surgery were excluded. The unexposed group received no surgery for morbid obesity and related comorbidities (no surgery group). The bariatric surgery group underwent the procedure at 18 years or older during the study period and used either lipid-lowering, cardiovascular, or antidiabetic medication during a 6-month period prior to surgery. The surgical management of obesity guidelines were identical in both countries throughout the study. Gastric bypass and sleeve gastrectomy were identified using country-specific procedural codes in the patient registries (eTable in Supplement 1). For each bariatric surgery patient, 5 controls in the no surgery group were exactly matched without replacement for country, age, sex, calendar year, and medication use (lipid-lowering, cardiovascular, antidiabetic medication, or any combination of these 3 medications 6 months prior to study entry). The matching ratio of 5:1 was chosen to maximize the statistical power and to enable exact matches within the limited pool of control patients. The management of obesity in the no surgery group could include, for example, physiotherapy, psychologist or dietician interventions, counseling, treatment of comorbid conditions, dietary modifications, and medications, but there was no standardized management protocol. Furthermore, the time point of medical intervention could not be assessed for patients in the no surgery group.

### Outcomes

The outcomes were changes in cardiovascular, lipid-lowering, or diabetic medication during the follow-up. These medication groups were defined from the Anatomical Therapeutic Chemical codes: lipid-lowering medication included all medications with Anatomical Therapeutic Chemical codes C10, eg, statins, fibrates, and other lipid-lowering agents; cardiovascular medication included all medications with Anatomical Therapeutic Chemical codes C01-C04, C07-C09, eg, cardiac glycosides, antiarrhythmics, antihypertensives, diuretics, peripheral vasodilators, β-blockers, calcium channel blockers, and renin-angiotensin system affecting drugs; and antidiabetic medication included all medications with Anatomical Therapeutic Chemical codes A10, eg, insulins, metformin, sulfonylureas, dipeptidyl peptidase-4 inhibitors, and sodium-glucose co-transporter 2 inhibitors. The time window of medication use was defined as the time from the date of dispensation of the relevant drug until the number of dispensed defined daily doses for that drug had passed up to a maximum of 100 days. If a new dispensation occurred during this period, the window of medication use was expanded to include the newly prescribed defined daily doses. A patient was considered a medication user at any given month after study entry if the time window for medication use included parts of or the entire month. If the month was outside the window of medication, the patient was considered a nonuser. Participants could change status several times. The outcomes were calculated also for the year before study entry.

### Statistical Analysis

The bariatric surgery patients entered the study at the date of surgery, and patients in the no surgery group as defined by the matching process. All patients were followed up until death or end of follow-up (December 31, 2018, in Finland and December 31, 2020, in Sweden), whichever occurred first. There were no limits specified for the length of follow-up. Because there were very little missing data, a complete case analysis was conducted. The proportion of users was presented as frequencies and plotted monthly for each of the 2 comparison groups before and after inclusion using a lowess smoothing with 0.1 bandwidth.^15^ Longitudinal analyses used multilevel mixed-effects logistic regression models with identifier variable as random effect. The standard errors were derived considering the intragroup correlation within matching groups. To plot the trajectories of medication along with 95% CIs from 1 year before study entry to 23 years after study entry by exposure, the fixed part of the mixed model included the exposure, cubic splines of time (4 knots at 0, 2, 5, 10, and 15 years), and the interaction term between exposure and time. The Wald tests for the 4 interaction terms was statistically significant (*P* < .001). For temporal patterns of medication use, the rates of change in odds of medication use over time for both groups were analyzed and presented as odds ratios (OR) with 95% CI. In a crude model (model 1) of the multilevel mixed regression, the exposure (bariatric surgery), the time variable modeled with linear spline with nodes at 2 and 10 years, and the interaction between the time and exposure variables were treated as fixed effects. In model 2, model 1 was further adjusted for comorbidity (Charlson Comorbidity Index 0 [reference], 1, or ≥2). Comorbidity was not matched for to avoid multicollinearity issues and to restrict the number of matching variables. Charlson Comorbidity Index score (the most well-validated version) was calculated for each patient based on diagnosis codes recorded in the patient registries.^16^ Model 3 included the exposure, the linear splines of time, the variable country, and a 3-way interaction between the exposure, time, and country. From these 3 models, the yearly rates of change in odds of medication use (slope of the curve of change) were calculated stratified for the time intervals 0 to 2, more than 2 to 10, and more than 10 years. Lastly, the ORs with 95% CI of drug dispensation stratified for the prespecified time intervals 0 to 1, more than 1 to 3, more than 3 to 5, more than 5 to 10, more than 10 to 15, and more than 15 to 20 years comparing the bariatric surgery group and the matched group who did not undergo operation were calculated using mixed-effects regression. The fixed effect part included the exposure, the categorical time variable categorized as above, and the interaction between the exposure and time variable. All data management and statistical analyses were carried out according to a detailed a priori study protocol by a senior biostatistician (G.S.) using Stata/MP version 15.1 (StataCorp). Analysis took place between July 2021 and January 2022.

## Results

### Patients

The study included 26 396 patients who underwent bariatric surgery (with gastric bypass or sleeve gastrectomy) and 5 times as many (n = 131 980) matched control patients with morbid obesity treated with no surgery. The exclusions leading to these numbers are shown in eFigure 1 and eFigure 2 in Supplement 1. Characteristics of the study participants are presented in Table 1. The majority of patients came from Sweden (bariatric surgery: 20 527 [77.8%] vs no surgery 102 635 [77.8%]) and were women (bariatric surgery: 17 521 [66.4%] vs no surgery: 87 605 [66.4%]), and the median (IQR) age was 50 (43-56) years. The median (range) follow-up was 7.1 (0-23) years in the bariatric surgery group and 5.7 (0-23) years in the no surgery group.

**Table 1.** Characteristics of Patients With Obesity Undergoing Bariatric Surgery and No Such Surgery in Sweden or Finland

Characteristic	No. (%)	
	Bariatric surgery (n = 26 396)	No surgery (n = 131 980)
Age, median (IQR), y	50 (43-56)	50 (43-56)
Sex		
Male	8875 (33.6)	44 375 (33.6)
Female	17 521 (66.4)	87 605 (66.4)
Calendar year, median (IQR)	2013 (2011-2016)	2013 (2011-2016)
Country		
Sweden	20 527 (77.8)	102 635 (77.8)
Finland	5869 (22.2)	29 345 (22.2)
Charlson Comorbidity Index, median (IQR)	1 (0-2)	1 (0-2)
Medication use before entry for		
High lipids only^a^	779 (3.0)	3895 (3.0)
Cardiovascular disease only^a^	12 241 (46.4)	61 205 (46.4)
Diabetes only^a^	2125 (8.1)	10 625 (8.1)
High lipids and cardiovascular disease only^a^	2761 (10.5)	13 805 (10.5)
High lipids and diabetes only^a^	754 (2.9)	3770 (2.9)
Cardiovascular and diabetes only^a^	3319 (12.6)	16 595 (12.6)
High lipids, cardiovascular disease, and diabetes	4417 (16.7)	22 085 (16.7)
High lipids^b^	8711 (33.0)	43 555 (30.0)
Cardiovascular disease^b^	22 738 (86.1)	113 960 (86.1)
Diabetes^b^	10 615 (40.2)	53 075 (40.2)
Follow-up, median (IQR), y	7.1 (4.1-9.6)	5.7 (2.5-8.6)

^a^ Not using the other medication group(s).

^b^ Without or with any combination with the other medication group(s).

### Lipid-Lowering Medication

**Figure 1.**
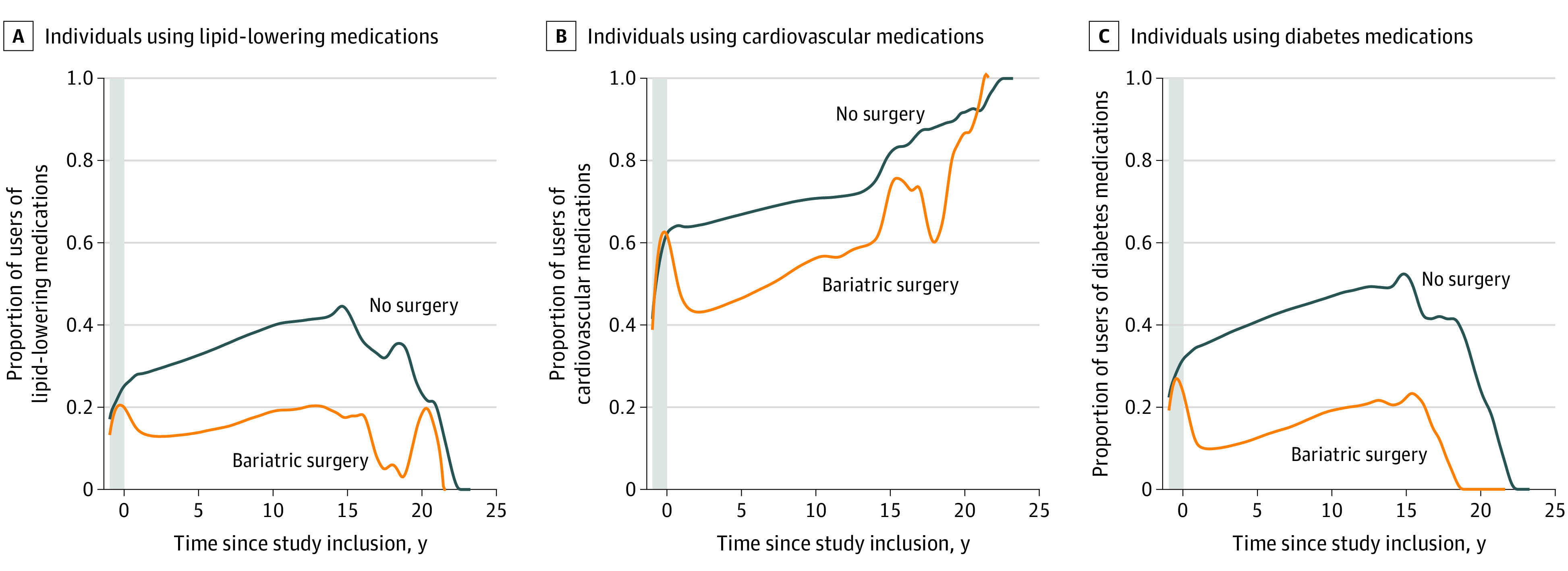
Proportions of Users of Lipid-Lowering, Cardiovascular, and Antidiabetic Medications Among Patients Treated With Bariatric Surgery and No Surgery for Obesity The gray band represents the year prior to inclusion.

At baseline (1 year prior to inclusion), 20.3% (95% CI, 20.2%-20.5%) of the bariatric surgery patients used lipid-lowering medication. This proportion declined to 12.9% (95% CI, 12.7%-13.0%) 2 years after surgery (difference, −7.5% [95% CI, −7.3% to −7.7%] compared with baseline). Thereafter, the proportion increased to 14.1% (95% CI, 14.0%-14.3%) after 5 years (difference, −6.2% [95% CI, −6.0% to −6.4%] compared with baseline) and 17.6% (95% CI, 13.3%-21.8%) after 15 years (difference, −2.8% [95% CI, 1.5% to −7.1%] compared with baseline; Figure 1A). In the no surgery group, the proportion of lipid-lowering drug users continuously increased over time from 21.0% (95% CI, 20.9%-21.1%) at baseline to 44.6% (95% CI, 41.7%-47.5%) after 15 years of follow-up (difference, 23.6% [95% CI, 20.7%-26.5%]; Figure 1A). The difference in differences in lipid-lowering medication comparing bariatric surgery with no surgery were −15.9% (95% CI, −15.6% to −16.2%) at 2 years, −20.0% (95% CI, −19.7% to −20.3%) at 5 years, and −26.4% (95% CI, −21.2% to −31.6%) at 15 years.

### Cardiovascular Medication

At baseline, 60.2% (95% CI, 60.0%-60.5%) of the bariatric surgery patients used cardiovascular medication. The use decreased to 43.2% (95% CI, 42.9%-43.4%) after 2 years (difference, −17.1% [95% CI, −16.7% to −17.4%] compared with baseline) and increased to 47.1% (95% CI, 46.8%-47.4%) after 5 years (difference, −13.2% [95% CI, −12.8% to −13.6%] compared with baseline) and increased further to 74.6% after 15 years (95% CI, 65.8%-83.4%; difference, 14.4% [95% CI, 5.6%-23.2%] compared with baseline; Figure 1B). In the no surgery group, the proportion of users of cardiovascular medication increased throughout the follow-up from 54.4% (95% CI, 54.3%-54.5%) at baseline to 83.3% (95% CI, 79.3%-87.3%) after 15 years (difference, 28.9% [95% CI, 24.9%-33.0%] compared with baseline; Figure 1B). The difference in differences in cardiovascular medication comparing bariatric surgery to no surgery were −27.2% (95% CI, −26.8% to −27.6%) at 2 years, −28.0% (95% CI, −27.5% to −28.5%) at 5 years, and −14.6% (95% CI, −4.9% to −24.3%) at 15 years.

### Antidiabetic Medication

At baseline, 27.7% (95% CI, 27.6%-27.9%) of bariatric surgery patients used antidiabetic medication. The proportion of users declined to 10.0% (95% CI, 9.9%-10.2%) 2 years after surgery (difference, −17.7% [95% CI, −17.7% to −17.9%] compared with baseline), after which the proportion increased to 13.1% (95% CI, 12.9%-13.2%) after 5 years (difference, −14.7% [95% CI, −14.4% to −14.9%] compared with baseline), and 23.5% (95% CI, 18.5%-28.5%) after 15 years of follow-up (difference, −4.2% [95% CI, −0.7% to −9.2%] compared with baseline; Figure 1C). In the no surgery group, the use of antidiabetic medication increased throughout the follow-up, from 27.7% (95% CI, 27.6%-27.7%) at baseline to 54.2% (95% CI, 51.0%-57.5%) after 15 years (difference, 26.6% [95% CI, 23.3%-29.8%] compared with baseline; Figure 1C). The difference in differences in antidiabetic medication comparing bariatric surgery with no surgery were −27.2% (95% CI, −26.7% to 27.2%) at 2 years, −29.9% (95% CI, −29.6% to −30.2%) at 5 years, and −30.8% (95% CI, –24.9% to −36.7%) at 15 years.

### Lipid-Lowering Medication, Cardiovascular Medication, or Antidiabetic Medication

**Figure 2.**
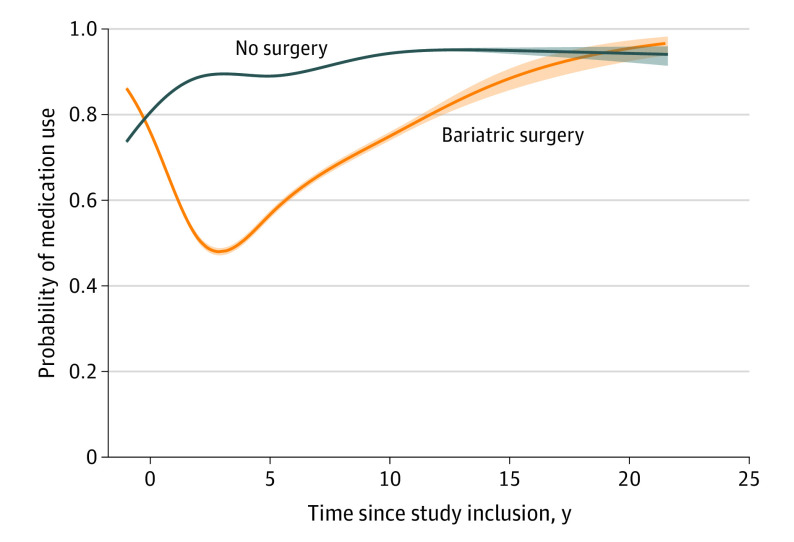
Temporal Changes in the Probability of Using Lipid-Lowering, Cardiovascular, or Antidiabetic Medications Among Patients Treated With Bariatric Surgery and No Surgery for Obesity The lines represent the point estimates and the shaded areas, 95% CIs.

The use of either lipid-lowering medication, cardiovascular medication, or antidiabetic medication decreased in the bariatric surgery group during the time interval 0 to 2 years after surgery (OR, 0.43 per year [95% CI, 0.42-0.44]), after which it increased during the time intervals 2 to 10 years (OR, 1.21 per year [95% CI, 1.20-1.22]), and more than 10 years after surgery (OR, 1.14 per year [95% CI, 1.09-1.20]) (Table 2). The no surgery group had a decrease in medication use between 0 and 2 years after inclusion (OR, 0.86 per year [95% CI, 0.85-0.87]) and an increase in the periods 2 to 10 years (OR, 1.12 per year [95% CI, 1.12-1.13]) and more than 10 years after inclusion (OR, 1.13 per year [95% CI, 1.10-1.16]) (Table 2). The decrease in OR of medication use was stronger in the bariatric surgery group compared with the no surgery group within the first 2 years (OR, 0.50 per year [95% CI, 0.48-0.51]), and the increase was stronger within 2 to 10 years in the bariatric surgery group (OR, 1.08 per year [95% CI, 1.07-1.09]), while no difference remained after more than 10 years (OR, 1.01 per year [95% CI, 0.96-1.07]) comparing the bariatric surgery group with the no surgery group (Table 2). The probability of dispensing a prescription for lipid-lowering, cardiovascular, or diabetic medication was lower in the bariatric surgery group compared with the no surgery group in all follow-up periods (Table 3 and Figure 2), with ORs of 0.32 (95% CI, 0.31-0.33) after 0 to 1 year and 0.22 (95% CI, 0.20-0.24) after 10 to 15 years.

**Table 2.** Yearly Changes in Probability of Lipid-Lowering, Cardiovascular, or Antidiabetic Medication in Patients With Obesity Undergoing Bariatric Surgery and No Such Surgery, Stratified by Follow-up Periods

Model	Odds ratio (95% CI)		
	Bariatric surgery	No surgery	Difference in change bariatric surgery vs no surgery
Model 1^a^			
0-2 y	0.43 (0.42-0.44)	0.86 (0.85-0.87)	0.50 (0.48-0.51)
3-10 y	1.21 (1.20-1.22)	1.12 (1.12-1.13)	1.08 (1.07-1.09)
>10 y	1.14 (1.09-1.20)	1.13 (1.10-1.16)	1.01 (0.96-1.07)
Model 2^b^			
0-2 y	0.43 (0.42-0.44)	0.86 (0.85-0.87)	0.50 (0.48-0.51)
3-10 y	1.21 (1.20-1.22)	1.12 (1.12-1.13)	1.08 (1.07-1.09)
>10 y	1.14 (1.09-1.20)	1.13 (1.10-1.16)	1.01 (0.96-1.07)
Model 3^c^			
Sweden			
0-2 y	0.42 (0.39-0.44)	0.93 (0.91-0.96)	0.45 (0.42-0.47)
3-10 y	1.25 (1.22-1.28)	1.13 (1.11-1.14)	1.11 (1.08-1.14)
>10 y	1.00 (0.81-1.23)	1.17 (1.07-1.29)	0.85 (0.69-1.05)
Finland			
0-2 y	0.43 (0.42-0.44)	0.85 (0.84-0.86)	0.51 (0.49-0.52)
3-10 y	1.21 (1.20-1.22)	1.12 (1.12-1.13)	1.08 (1.07-1.09)
>10 y	1.17 (1.12-1.22)	1.13 (1.09-1.16)	1.04 (0.99-1.09)

^a^ Model 1: patient identifier and exchangeable covariance as random effects and exposure, time, and 2-way interaction as fixed effects.

^b^ Model 2: same as model 1 but also adjusted for comorbidity.

^c^ Model 3: patient identifier and exchangeable covariance as random effects and exposure, time, country, and 3-way interaction as fixed effects, and adjusted for comorbidity.

**Table 3.** Probability of Lipid-Lowering, Cardiovascular, or Antidiabetic Medication Comparing Patients With Obesity Undergoing Bariatric Surgery and No Such Surgery, Stratified by Follow-up Periods

Year	Odds ratio (95% CI)	
	No surgery	Bariatric surgery
1-0	1 [Reference]	1.88 (1.83-1.93)
0-1	1 [Reference]	0.32 (0.31-0.33)
2-3	1 [Reference]	0.15 (0.15-0.16)
4-5	1 [Reference]	0.14 (0.14-0.15)
6-10	1 [Reference]	0.17 (0.17-0.18)
11-15	1 [Reference]	0.22 (0.20-0.24)
16-23	1 [Reference]	0.11 (0.03-0.42)
*P* value for trend	<.001	

## Discussion

This study suggests that patients who undergo bariatric surgery have a long-term reduction in the use of lipid-lowering and antidiabetic medications compared with matched control patients who did not undergo operation with obesity diagnosis, while the reduction was only transient for cardiovascular medications.

Only 2 fairly large studies have compared the use of obesity-related medications in patients having undergone bariatric surgery with control patients who did not undergo operation. A multicenter study of individuals with type 2 diabetes from the United States, including 2287 bariatric surgery patients and 11 435 matched controls, found reduced use of lipid-lowering, cardiovascular, and antidiabetic medications after bariatric surgery.^2^ A French population-based nationwide study with 8199 bariatric surgery patients and controls suggested a stronger reduction in antihypertensive medication and lipid-lowering medication after bariatric surgery.^7^ However, in contrast to the present study, both of these studies had smaller sample sizes, shorter follow-up time (up to 8 and 6 years, respectively), did not match controls for medication use, and included nonusers of medications, which might bias the estimates of medication resolution after bariatric surgery. Furthermore, a Swedish cohort study of 2010 patients undergoing bariatric surgery (68% vertical banded gastroplasty, 19% gastric banding, and 13% gastric bypass) and 2037 controls suggested lower medication costs after bariatric surgery during 20-year follow-up. However, the proportions of medication use were not reported over time, and most patients in the bariatric surgery group underwent procedures that are not in clinical use currently.

The use of lipid-lowering, cardiovascular, and antidiabetic medications was markedly reduced during the first years of follow-up after bariatric surgery compared with baseline in the present study. However, a slow increase in lipid-lowering and cardiovascular medication was observed over time, exceeding baseline levels for lipid-lowering and cardiovascular medications after long follow-up, while antidiabetic medication was persistently lower than baseline. These changes may be related to aging and regain of weight over time after bariatric surgery, a phenomenon caused by hormonal, dietary, physical, and behavioral factors.^17^ In contrast, and as expected, a gradual increase in the use of all 3 medication groups was observed over time among the patients treated with no surgery for obesity. After 15 years of follow-up, medication use in the bariatric surgery group exceeded that of the control group. However, the estimates after 15 years of follow-up should be interpreted cautiously due to low power and potential selection due to competing risks during the long follow-up. On the other hand, the results are in line with a US study (n = 95 405) showing that bariatric surgery results in discontinuation of lipid-lowering medications in over 60% of patients, antihypertensives in over 50%, and diabetes medications in over 70% of patients within 5 years of surgery.^18^ However, more than 40% restarted lipid-lowering medication, more than 65% restarted cardiovascular medication, and more than 30% restarted diabetes medication.^18^

The results can aid in informed decision-making when considering bariatric surgery for patients with morbidly obesity and inform patients and professionals about the expected long-term effects of medication use for obesity-related comorbidities. Economically, the long-lasting reductions in medication use for hyperlipidemia, cardiovascular morbidity, and diabetes suggest that surgical treatment of morbid obesity may infer savings in medication expenses for patients, health care, and society. Future research may focus on subgroups that are most likely to benefit from bariatric surgery, including resolution and severity of comorbidities.

### Strengths and Limitations

Among strengths of the study are the long and complete follow-up, population-based design, matching of comparable control patients with morbid obesity, and the large sample size retrieved from 2 countries. The study periods of 1995 to 2018 in Finland and 2005 to 2020 in Sweden were dependent on the initiation years of medication registries. This difference might induce temporal bias. However, the clinical criteria and technique of gastric bypass and sleeve gastrectomy were similar during the study period, and other types of bariatric surgery were excluded. Thus, any temporal bias should not be a major concern. In the no surgery group, information on the time point or methods of obesity treatment was not available. It could be argued that more comorbid patients are included in the no surgery group than in the bariatric surgery group, or vice versa. However, exact matching for lipid-lowering, cardiovascular, and antidiabetic medications should alleviate this concern, although the doses of medications could still differ between the groups. All medications under study require prescriptions and were thus captured by the medication registries. Dispensation of medications may not always mean taken medications, but it is difficult to control for in this type of study design. However, it is unlikely that patients would dispense medications for several months or years without taking any. Furthermore, medication cessation is not equal to disease resolution, and therefore making conclusions on disease resolution based on these data should be avoided. The registry data used have been validated for high quality and completeness. Yet, misclassification might still exist but should be nondifferential and only dilute differences between the groups and not explain them. Despite matching or adjusting for key variables, there could still be residual or unknown confounding with an observational design. Confounding by differences in severity of obesity or comorbidity in the operated and nonoperated patients cannot be outruled. However, as all had a diagnosis of obesity (indicating substantial obesity), the comorbidities were adjusted for, and the matching for medication use should reduce confounding. As another limitation, data on some potentially relevant variables were not available, eg, body mass index, smoking, alcohol use, and socioeconomic status. However, these variables were to some extent indirectly adjusted for in the matching process and the Charlson Comorbidity Index score. It would have been valuable to stratify the results by body mass index if such data were available, but the matching for medication use should counteract bias. Despite the large cohort, the statistical power was limited in some subgroup analyses, particularly after 15 years of follow-up.

## Conclusions

In conclusion, this large population-based cohort study in Sweden and Finland with up to 23 years of follow-up indicates a long-lasting reduction in the use of lipid-lowering and antidiabetic medication in patients who undergo bariatric surgery compared with matched patients with an obesity diagnosis treated without surgery, while the reduction was only transient for cardiovascular medications.

## Notes

Supplement 1. eTable. Diagnosis codes according to International Classification of Diseases (ICD) versions 10 and 9 and surgical codes used for identification of study participants eFigure 1. Selection of patients having undergone bariatric surgery in Sweden or Finland (bariatric surgery group) eFigure 2. Formation of a control group of patients with no surgery for obesity matched to patients having undergone bariatric surgery in Sweden or Finland (no surgery group)

Supplement 2. Data sharing statement
