# Supplementary material for: Temporal Changes in Obesity-Related Medication After Bariatric Surgery vs No Surgery for Obesity
Source: JAMA Surg. 2023 May 24;158(8):817–23. doi: 10.1001/jamasurg.2023.0252 (PMC10209832; doi:10.1001/jamasurg.2023.0252)
Supplement: Supplement 1. — eTable. Diagnosis codes according to International Classification of Diseases (ICD) versions 10 and 9 and surgical codes used for identification of study participants eFigure 1. Selection of patients having undergone bariatric surgery in Sweden or Finland (bariatric surgery group) eFigure 2. Formation of a control group of patients with no surgery for obesity matched to patients having undergone bariatric surgery in Sweden or Finland (no surgery group) [file jamasurg-e230252-s001.pdf]

Supplemental Online Content

Kauppila JH, Markar S, Santoni G, Holmberg D, Lagergren J. Temporal changes in obesity-related medication after bariatric surgery vs no surgery for obesity. *JAMA Surg*. Published online May 24, 2023.
doi:10.1001/jamasurg.2023.0252

eTable. Diagnosis codes according to International Classification of Diseases (ICD) versions 10 and 9 and surgical codes used for identification of study participants
eFigure 1. Selection of patients having undergone bariatric surgery in Sweden or Finland (bariatric surgery group)
eFigure 2. Formation of a control group of patients with no surgery for obesity matched to patients having undergone bariatric surgery in Sweden or Finland (no surgery group)

This supplemental material has been provided by the authors to give readers additional information about their work.

© 2023 Kauppila JH et al. *JAMA Surgery.*

eTable. Diagnosis codes according to International Classification of Diseases (ICD) versions 10 and 9 and surgical codes used for identification of study participants.

**Obesity diagnosis**	**ICD-10 code**	**ICD-9 code**
	E66	278A, 278.0
**Bariatric surgery**	**NOMESCO-codes**	**Surgery codes in Sweden before 1996**
Gastric bypass	JDF10, JDF11	4752
Sleeve gastrectomy	JDF41, JDF96,<br>JDF97 after year 2000<br>JDF00 after year 2000<br>JDF01 after year 2000	

© 2023 Kauppila JH et al. *JAMA Surgery.*

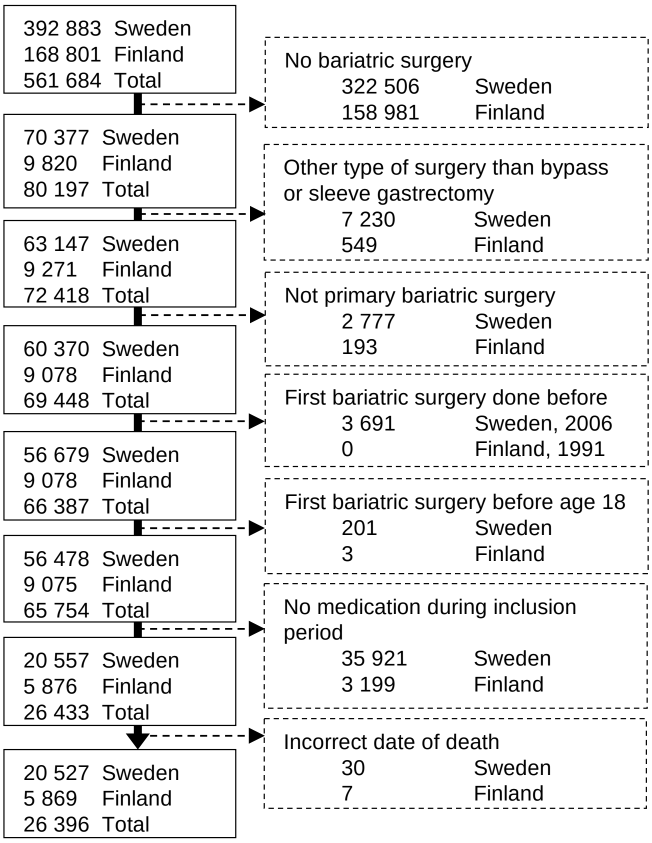

eFigure 1. Selection of patients having undergone bariatric surgery in Sweden or Finland (bariatric surgery group).

© 2023 Kauppila JH et al. *JAMA Surgery.*

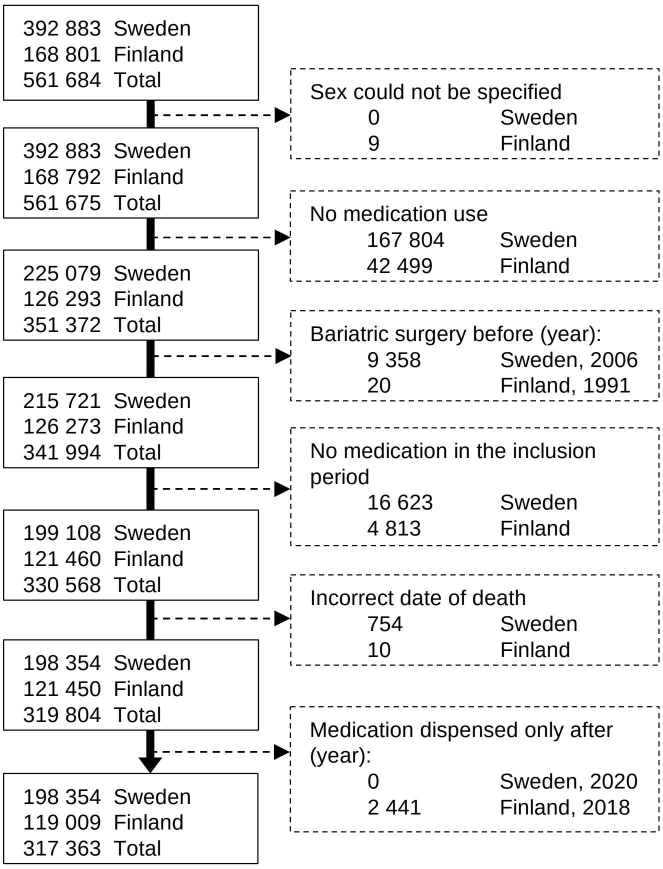

eFigure 2. Formation of a control group of patients with no surgery for obesity matched to patients having undergone bariatric surgery in Sweden or Finland (no surgery group).

© 2023 Kauppila JH et al. *JAMA Surgery.*
