# Supplementary material for: Temporal Changes in Obesity-Related Medication After Bariatric Surgery vs No Surgery for Obesity
Source: JAMA Surg. 2023 May 24;158(8):817–23. doi: 10.1001/jamasurg.2023.0252 (PMC10209832; doi:10.1001/jamasurg.2023.0252)
Supplement: Supplement 2. — Data sharing statement [file jamasurg-e230252-s002.pdf]

Data Sharing Statement

Kauppila. Temporal Changes in Obesity-Related Medication After Bariatric Surgery vs No Surgery for Obesity. *JAMA Surg*. Published May 24, 2023. doi:10.1001/jamasurg.2023.0252

Data
Data available: No

Additional Information
Explanation for why data not available: The data can be shared for research purposes upon request by contacting the Chief Investigator, Professor Jesper Lagergren, but may be restricted by and require complimentary permissions from the relevant ethical committees and original data holders.
